# Characteristics and trends of childhood cancer in Pudong, China, 2002–2015

**DOI:** 10.1186/s12889-020-09493-9

**Published:** 2020-09-21

**Authors:** Junqi Ji, Zheng Luo, Yichen Chen, Xiaoyun Xu, Xiaopan Li, Shijian Liu, Shilu Tong

**Affiliations:** 1 Department of Clinical Epidemiology and Biostatistics, Children Health Advocacy Institute, Shanghai Children’s Medical Center, Shanghai Jiao Tong University School of Medicine, 1678 Dongfang Road, Shanghai, 200127 China; 2 Shanghai University of Medicine & Health Sciences Affiliated Zhoupu Hospital, Shanghai, China; 3 Pudong New Area Center for Disease Control and Prevention, Fudan University Pudong Institute of Preventive Medicine, Pudong New Area, Shanghai, China; 4 School of Public Health, Shanghai Jiao Tong University School of Medicine, Shanghai, China; 5 School of Public Health, Institute of Environment and Population Health, Anhui Medical University, Hefei, China; 6 Center for Global Health, School of Public Health, Nanjing Medical University, Nanjing, China; 7 School of Public Health and Social Work, Queensland University of Technology, Brisbane, Australia

**Keywords:** Childhood cancer, Incidence rate, Survival rate, Population-based cancer registry, Time trends

## Abstract

**Background:**

With the growing threat of cancer to children’s health, it is necessary to analyze characteristics and trends of childhood cancer to formulate better cancer prevention strategies.

**Methods:**

Data on the 430 children with cancer during 2002–2015 were collected from the Pudong Cancer Registry, diagnosed with the International Classification of Diseases for Oncology and categorized according to the International Classification of Childhood Cancer. The incidence rate, trends over time, and survival of patients grouped by sex, age, and region were explored using the Kaplan-Meier, Cox regression, and Joinpoint Regression models.

**Results:**

The crude childhood cancer incidence and world age-standardized incidence rate (ASR) were 115.1/1,000,000 and 116.3/1,000,000 person-years. The two most frequent cancers were leukemia (136/430, 31.63%, ASR, 37.8/1,000,000 person-years) and central nervous system (CNS) tumors (86/430, 20.00%, ASR, 22.9/1,000,000 person-years). Our findings indicate that the survival rate for children between 10 and 15 years of age was higher than that for 5–10; and the survival rate for children who had leukemia was higher than that of children with CNS tumors. However, the overall incidence of childhood cancer, and leukemia, CNS tumors remained relatively stable in Pudong between 2002 and 2015.

**Conclusions:**

The incidence and survival rate for childhood cancer patients varied by age and cancer type. The overall trends of childhood cancer incidence remained relatively stable in Pudong from 2002 to 2015 even though socioeconomic development has been unprecedentedly fast in this region.

## Background

The occurrence of cancer in children is a heavy blow not only for themselves, but also for their families. Although the mortality pattern of childhood cancer has changed in the majority of developed countries and survival rates have significantly improved due to advanced medical technology, it remains the second most common cause of death (following accidents) for the pediatric population [1]. Describing the epidemiology of childhood cancer can improve our understanding on cancer etiology and promote the critical assessment of current protocols for cancer control and prevention [2]. The Surveillance, Epidemiology, and End Results (SEER) program in the United States and the Automated Childhood Cancer Information System in Europe has closely monitored the epidemiology of childhood cancer and reported increasing trends in childhood cancer incidence from 1974 to 2014 and from 1978 to 1997 [3–5]. However, limited data is available on the characteristics and trends of childhood cancer in China, particularly for rapidly developing areas like Pudong.

In the past forty years of reform and opening-up, China’s total economic output has increased by more than 200 times. China’s share of global economic output has risen from 2 to 15% [6]. Pudong, an important district which is located in the southeast of Shanghai, with urban and suburban, is a symbol of China’s reform. Specifically, the gross domestic product (GDP) in Pudong has increased from 10.1 billion RMB in 1992 to 789.8 billion RMB in 2015, with an average annual growth rate of 15.6%. Since Pudong merged previous Nanhui district in 2009, so the whole metropolitan area of Pudong has risen from 517.83 km^2^ in 1995 (8.12% of the land area of Shanghai) to 1373.82 km^2^ in 2015 (21.67% of Shanghai). The permanent population has increased from 2.40 million in 2000 (14.92% of Shanghai) to 5.47 million in 2015 (22.7% of Shanghai) [7]. Social and economic development is widely regarded as an essential factor for the improvement of people’s health [8, 9]. In fact, cancer has been ranked as the second leading cause of death in Pudong since 1993 [10]. With the development of Pudong, residents are more concerned with the risk factors of diseases such as cancer. Moreover, with the release of the government “second child” policy in 2016, parents pay more attention to prenatal and postnatal care as well as environmental quality. As the epitome of China’s development, Pudong is a unique and valuable place for analyzing characteristics and trends of childhood cancer and exploring effective strategies to control and prevent this disease.

The aims of this study are threefold: firstly, to examine the overall cancer incidence in children from 2002 to 2015 and the characteristics of childhood cancer; secondly, to study the trends of childhood cancer overtime and by different cancer type; finally, to explore any changes in survival rates for children with cancer.

## Methods

### Study participants

The participants were the children under 15-year-old residing in Pudong during 2002–2015 (registered at birth in Pudong). Childhood cancer diagnoses were coded according to the International Classification of Diseases for Oncology [11], and categorized by cancer type and age group (0–14, including 0–4, 5–9, and 10–14 subgroups) according to the International Classification of Childhood Cancer (ICCC-3) [12].

### Data collection

The diagnostic information was collected from the Pudong Cancer Registry from 2002 to 2015. The number of urban and suburban areas covered by Pudong Cancer Registry has increased since Pudong new district was formed after both the previous Nanhui district and old Pudong district merged in 2009 [13, 14]. Pudong Cancer Registry collects, evaluates, and publishes cancer data reported from local hospitals and community health centers as well as the Urban Resident Basic Medical Insurance program and the New Rural Cooperative Medical Scheme. Patients who gave informed consent and accepted the community doctors’ survey were followed. Follow-up entailed household survey was conducted by telephone calls every year according to our standard epidemiologic procedure. The survival information of patients lost to follow-up was obtained from the coroner’s registrar restricted to residents of the metropolitan area of Shanghai. The quality of submitted data was checked and evaluated based on the *Guidelines for Chinese Cancer Registration* [15] and International Agency for Research on Cancer/International Association of Cancer Registries (IARC/IACR) data-quality criteria [16]. Population data were provided by the Statistics Bureau and the Public Security Bureau of Pudong. In addition, we cited and compared the childhood cancer incidence of other six population-based cancer registries (Shanghai, Beijing, Guangzhou, Hongkong, Dalian, Zhongshan) in China from the International Incidence of Childhood Cancer (IICC) project (http://iicc.iarc.fr/results/comparative.php).

### Statistical analysis

Cancer incidence and its world age-standardized rate (ASR) were calculated based on the annual average population in Pudong and world standard population, respectively. Chi-square tests were utilized to examine the difference between cancer incidences. The annual percentage change was used to analyze the trend of cancer incidence over time. Multivariate Cox regression model was applied to analyze survival risk factors, and overall survival rate was analyzed using the Kaplan-Meier method. The log-rank test was used to compare survival curves. The statistical analysis was performed using the statistical software Stata (version 14.0, Stata corp, College Station, TX) and Joinpoint Regression Program (version 4.0.4, National Cancer Institute, Bethesda, MD) [17]. Statistical significance was set at *P*-value < 0.05 on both sides.

## Results

### Characteristics

Specifically, the number of registered cancer patients was 430 with a mean age 7-year-old; the number of female patients (179, 41.63%) was less than male patients (251, 58.37%); 148 (34.42%) of them were from urban areas (12 sub-districts), whereas 65.58% (282) of them were from suburban areas (24 towns). From 2002 to 2015, 430 childhood cancer cases were registered, accounting for 2.53% of all cancer cases in Pudong, the epitome of China’s development and accounted for one fifth of Shanghai’s geography and population. According to the summary statistics shown in Table 1, the seven common childhood cancers in accordance to descending order of case number were leukemia (136, 31.63%), CNS tumors (86, 20.00%), bone and articular cartilage cancer (29, 6.74%), lymphoma (29, 6.74%), endocrine tumors (22, 5.12%), and renal carcinoma (19, 4.42%). Specifically, the number of leukemia cases was highest for children under 5-year-old and decreased as the age increased. Also, leukemia was more common in male than female, with a gender ratio of 1.39. Moreover, the number of cases for CNS tumors was highest for children aged 5 to 10 and lowest for children aged under 5. CNS tumors were more common in male than in female as well, with a gender ratio of 1.21. The incidence of neuroblastoma apparently increased with the age. For the age group, as shown in Table 2, 170 (39.53%), 116 (26.97%) and 144 (33.49%) of them were under 5, aged between 5 and 10, and aged 10–15, respectively.

**Table 1:** Incidence of children cancer by cancer type in Pudong from 2002 to 2015

Type of tumors	Age							Percentage % 0–14	Incidence rate per million person-years		MV %	DCO %
	0 ≤ Age < 5		5 ≤ Age < 10		10 ≤ Age < 15		Total					
	Male	Female	Male	Female	Male	Female			0–14	ASR	0–14	0–14
I. Leukemia	32	32	28	11	19	14	136	31.6	36.4	37.8	86.8	0
II. Lymphoma	5	1	8	3	10	2	29	6.7	7.8	7.5	96.6	0.3
III. CNStumors	8	15	24	11	15	13	86	20.0	23.0	22.9	80.2	0.1
IV. Neuroblastoma	3	3	3	4	5	8	26	6.1	8.1	7.8	92.3	0
V. Retinoblastoma	0	3	1	0	1	4	9	2.1	0.2	0.2	100.0	0
VI. Renal tumors	8	7	1	2	1	0	19	4.4	5.1	5.7	100.0	0
VII. Hepatic tumors	0	4	0	1	1	2	8	1.9	2.2	2.4	100.0	0
VIII. Malignant bone tumors	0	0	4	2	15	8	29	6.7	7.8	6.8	93.1	0
IX. Soft-tissue and other extraosseous sarcomas	3	3	4	0	0	2	12	2.8	0.3	0.3	83.3	0
X. Germ cell and gonadal tumors	7	2	0	3	0	3	15	3.5	0.4	0.4	86.7	0
XI. Other malignant melanomas and epithelial neoplasm	0	2	0	0	0	1	3	0.7	0.5	0.5	66.7	0
XII. Other	30	4	5	0	11	8	58	13.5	24.4	24.2	89.7	0
Total	96	76	78	37	78	65	430	100.0	116.2	116.5	88.1	0.4

*CNS* central nervous system, *Other* gastric cancer, parotid carcinoma, mediastinal carcinoma, peritoneal cancer, gallbladder carcinoma, nasal carcinoma, appendiceal cancer and other malignant tumors, *ASR* age-standard rate, *DCO* death certificate only, *MV* microscopically verified

**Table 2:** Trends of cancer in children in Pudong from 2002 to 2015 (analyzed by Joinpoint Regression Program)

Characteristics	Number of Cases	Age specific annual percentage change	Lower limit	Upper limit	Z	P
Gender						
Male						
Age (years)						
0-	92	4.3	−4.0	13.4	−1.1	0.3
5-	78	1.6	−4.3	7.8	−0.6	0.6
10-	81	−2.1	−6.7	2.7	0.9	0.3
Subtotal	251	0.8	−2.5	−4.3	−0.6	0.7
Female						
Age (years)						
0-	78	−1.6	−8.8	6.2	0.5	0.7
5-	38	−0.8	−7.7	6.5	0.3	0.8
10-	63	−0.5	−8.2	7.8	0.2	0.9
Subtotal	179	−1.5	−6.3	3.5	0.7	0.5
Overall						
Age (years)						
0-	170	1.4	−5.3	−8.5	−0.4	0.7
5-	116	0.3	−4.4	−5.2	−0.1	0.9
10-	144	−1.2	−5.3	3.0	0. 7	0.5
Total	430	0	−3.5	3.6	0.1	0.9
Cancer Type						
Male						
Leukemia	79	1.3	−5.1	8.0	−0.4	0.7
CNS tumors	47	2.6	−7.1	13.3	−0.6	0.6
Female						
Leukemia	57	−1.2	−10.3	9.0	0.3	0.8
CNS tumors	39	−12.1	−29.6	9.7	1.3	0.2
Overall						
Leukemia	136	1.1	−4.4	6.9	−0.4	0.7
CNS tumors	86	−0.6	−7.5	6.8	0.2	0.9

*CNS* Central nervous system

### Incidence rate and ASR of cancer

The annual crude incidence rate and ASR of childhood cancer was 116.2 and 116.5 per 1,000,000 person-years, respectively. The overall crude incidence rate and the ASR for males were 131.6/1,000,000 and 132.6/1,000,000 person-years respectively. On the other hand, the crude incidence rate and the ASR for females were 97.8/1,000,000 and 99.3/1,000,000 person-years respectively. For age groups, the ASRs for patients aged less than 5, between 5 and 10, and between 10 and 15 were 52.5/1,000,000, 31.7/1,000,000, and 32.1/1,000,000 person-years, respectively. As shown in Table 1, the crude incidence rate of leukemia was 36.4/1,000,000 and its ASR was 37.8/1,000,000 person-years. The crude incidence rate and ASR of CNS tumors were 23.0/1,000,000 and 22.9/1,000,000 person-years, respectively. The ASR of retinoblastoma, soft-tissue sarcomas, germ cell tumors in Pudong was 2.1/1,000,000, 2.8/1,000,000, and 3.5/1,000,000 person-years, respectively. The percentage of cases that had information from death certificate only (DCO%) was 0.46% while the percentage of cases whose diagnoses were microscopically verified (MV%) was 88.13%.

### Survival rate

**Fig. 1:**
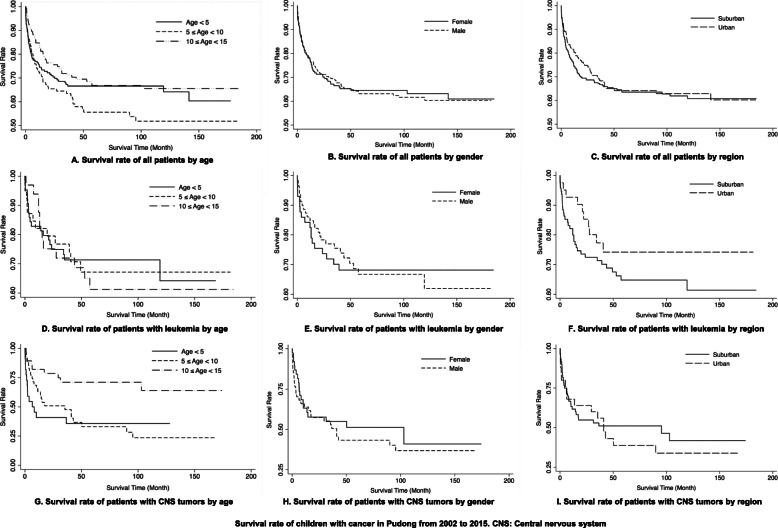
Survival rate of children with cancer in Pudong from 2002 to 2015. The curves show the survival rate of all patients by age (**a**), gender (**b**), region (**c**); the survival rate of patients with leukemia by age (**d**), gender (**e**), region (**f**); the survival rate of patients with tumors in central nervous system (CNS) by age (**g**), gender (**h**), region (**i**)

Figure 1 shows the survival rates of all patients and the survival rates of patients with leukemia and CNS tumors by gender, age, and region. As shown in Fig. 1 (a) (b) (c), the survival rate for children between 10 and 15 was higher than that for 5–10 (χ2 = 4.59, *P* = 0.034), but there was no statistically significant difference in survival rates based on gender or region. From Fig. 1 (d) (e) (f), we found no significant difference in survival for children with leukemia by gender, age, or region. As shown in Fig. 1 (g) (h) (i), the survival of children who had CNS tumors aged between 10 and 15 was higher than that for children aged 5–10 (χ2 = 10.71, *P* = 0.009) and that for children under 5-year-old (χ2 = 10.71, *P* = 0.005).

### Trend of incidence

Table 2 indicates the annual percentage change of childhood cancer by age and most frequent cancer types. There was no discernible difference in the overall incidence rate from 2002 to 2015.. Specifically, annual incidence rate increased by 1.08% for leukemia but decreased by 0.60% for CNS tumors. The annual percentage change of childhood cancer for different age groups was 1.38, 0.29%, and − 1.20% for aged less than 5, between 5 and 10, and between 10 to 15, respectively. However, none of the trend tests was statistically significant.

## Discussion

Like other diseases, the incidence of childhood cancer varies by population and geographic area. The ASR for childhood cancer in Pudong was 116.5/1,000,000 person-years from 2002 to 2015, lower than the ASR of China from 1990 to 2013 (131.9/1,000,000 person-years) and ranked in the middle compared to the six population-based cancer registries in China. Specifically, the ASR for childhood cancer in Pudong was lower than that of Beijing, Guangzhou, Dalian, and Hong Kong; but higher than that of Zhongshan and Shanghai [18] (Table 3). The reasons for geographic variation in the incidence of childhood cancer remain largely unknown.

**Table 3:** Comparison of children cancer by cancer registry in China

ICCC-3 group	IICC REGISTRY	ASR	%MV	%DCO
I. LEUKAEMIA	Pudong	37.8	86.8	0.0
	Beijing	50.1	99.9	0.0
	Dalian	54.1	100.0	0.0
	Guangzhou	59.2	98.4	0.0
	Hongkong	52.1	97.9	0.1
	Shanghai	38.5	97.8	0.3
	Zhongshan	29.4	99.3	0.0
	Combined 6 registries	49.1	98.7	0.1
II. LYMPHOMA & RELATED	Pudong	7.5	96.6	0.3
	Beijing	6.7	100.0	0.0
	Dalian	11.0	100.0	0.0
	Guangzhou	10.6	96.9	0.0
	Hongkong	11.3	90.7	0.0
	Shanghai	10.3	96.1	0.7
	Zhongshan	7.0	100.0	0.0
	Combined 6 registries	9.5	94.7	0.1
III. CNS NEOPLASMS	Pudong	22.9	80.2	0.1
	Beijing	20.5	68.4	0.8
	Dalian	20.1	60.0	11.4
	Guangzhou	23.3	80.5	1.8
	Hongkong	22.3	89.7	1.6
	Shanghai	23.5	70.6	3.3
	Zhongshan	19.4	81.9	0.0
	Combined 6 registries	21.6	78.6	1.9
IV. NEUROBLASTOMA	Pudong	7.8	92.3	0.0
	Beijing	8.4	100.0	0.0
	Dalian	3.0	100.0	0.0
	Guangzhou	8.1	96.6	0.0
	Hongkong	10.5	100.0	0.0
	Shanghai	9.4	100.0	0.0
	Zhongshan	2.5	100.0	0.0
	Combined 6 registries	8.1	99.8	0.0
V. RETINOBLASTOMA	Pudong	0.2	100.0	0.0
	Beijing	6.3	100.0	0.0
	Dalian	1.1	100.0	0.0
	Guangzhou	5.7	68.8	0.0
	Hongkong	6.7	100.0	0.0
	Shanghai	3.7	100.0	0.0
	Zhongshan	3.3	100.0	0.0
	Combined 6 registries	5.7	98.0	0.0
VI. RENAL TUMOURS	Pudong	5.7	100.0	0.0
	Beijing	5.4	98.8	0.0
	Dalian	6.9	91.7	0.0
	Guangzhou	3.3	90.0	0.0
	Hongkong	4.4	91.8	0.0
	Shanghai	2.9	100.0	0.0
	Zhongshan	6.2	95.0	0.0
	Combined 6 registries	4.7	95.0	0.0
VII. HEPATIC TUMOURS	Pudong	2.4	100.0	0.0
	Beijing	3.9	98.6	0.0
	Dalian	9.1	47.4	15.8
	Guangzhou	6.1	50.0	0.0
	Hongkong	5.5	86.6	0.6
	Shanghai	3.1	76.9	7.7
	Zhongshan	2.9	58.3	0.0
	Combined 6 registries	4.8	81.6	1.9
VIII. BONE TUMOURS	Pudong	6.8	93.1	0.0
	Beijing	5.0	89.2	0.5
	Dalian	3.7	87.5	8.3
	Guangzhou	4.6	95.2	0.0
	Hongkong	5.9	95.3	0.4
	Shanghai	4.8	96.6	0.0
	Zhongshan	3.2	96.3	0.0
	Combined 6 registries	5.2	93.2	0.6
IX. SOFT TISSUE SARCOMA	Pudong	0.3	83.3	0.0
	Beijing	5.8	100.0	0.0
	Dalian	2.9	100.0	0.0
	Guangzhou	7.9	100.0	0.0
	Hongkong	8.2	100.0	0.0
	Shanghai	5.9	100.0	0.0
	Zhongshan	2.6	100.0	0.0
	Combined 6 registries	6.6	100.0	0.0
X. GERM CELL TUMOURS	Pudong	0.4	86.7	0.0
	Beijing	5.6	99.0	0.0
	Dalian	3.2	94.4	0.0
	Guangzhou	10.1	98.2	0.0
	Hongkong	11.3	99.0	0.0
	Shanghai	6.4	94.9	0.0
	Zhongshan	5.1	100.0	0.0
	Combined 6 registries	8.3	98.5	0.0
XI. CARCINOMA & MELANOMA	Pudong	0.5	66.7	0.0
	Beijing	3.5	100.0	0.0
	Dalian	6.2	100.0	0.0
	Guangzhou	8.1	99.0	0.0
	Hongkong	4.4	99.8	0.0
	Shanghai	4.6	97.2	0.0
	Zhongshan	3.0	100.0	0.0
	Combined 6 registries	4.3	99.4	0.0
XII. OTHER & UNSPECIFIED	Pudong	24.2	89.7	0.0
	Beijing	3.1	19.8	7.7
	Dalian	21.2	17.0	35.8
	Guangzhou	2.5	25.0	8.3
	Hongkong	2.2	40.1	1.3
	Shanghai	1.3	60.0	13.3
	Zhongshan	3.6	65.0	0.0
	Combined 6 registries	3.2	35.2	9.3
TOTAL	Pudong	116.5	88.1	0.5
	Beijing	124.3	92.0	0.4
	Dalian	142.5	80.8	7.1
	Guangzhou	149.4	91.8	0.4
	Hongkong	144.6	94.1	0.3
	Shanghai	114.4	90.5	1.4
	Zhongshan	88.2	93.5	0.0
	Combined 6 registries	131.9	92.4	0.7

*ICCC-3* the third edition of International Classification of Childhood Cancer, *IICC* International Incidence of Childhood Cancer, *ASR* age-standard rate, *DCO* death certificate only, *MV* microscopically verified

The most frequent childhood cancer in Pudong was leukemia, followed by CNS tumors. The ASR of leukemia in Pudong was 37.8/1,000,000 person-years, lower than the ASR of leukemia in China and ranked as the second lowest compared to the six population-based cancer registries mentioned above. Slightly higher ASRs of leukemia were reported in Beijing, Dalian, Guangzhou, Hong Kong, and Shanghai, but Zhongshan had a slightly lower ASR. The ASR of CNS tumors in Pudong was very close to the ASR of CNS tumors in China and ranked in the middle compared to the six population-based cancer registries [18]. The ASR of retinoblastoma, soft-tissue sarcomas, germ cell tumors in Pudong was 2.1/1,000,000, 2.8/1,000,000, and 3.5/1,000,000 person-years, respectively, which was higher than that in Dalian, and lower than that in Beijing, Shanghai, Guangzhou, Hong Kong and Zhongshan. The DCO% was 0.5%, which was similar to that in Beijing, Guangzhou and Hong Kong, lower than that in Shanghai and Dalian, and higher than that in Zhongshan. In addition, the MV% was 88.1%, which was higher than that in Dalian and lower than that in other five registry cities. (Table 3) The incidence of neuroblastoma apparently increased with the age, the reverse of what is found in most populations.

Compared with adults, childhood cancer is relatively rare and diverse. Although many studies have been carried out to explore the etiology of childhood cancer, our understanding of its biological mechanism is still limited [19]. SEER program has listed specific risk factors for childhood cancer, including radiation, race and genetic factors For example, a study reports that protective maternal supplementation of folic acid can reduce the risk of B-cell acute lymphoblastic leukemia [20]; higher incidence of ALL is observed among children who live in newly painted houses after birth [21]; National Cancer Institute reports that father’s smoking can significantly increase the risk of cancer in children, especially acute leukemia and lymphoma [22]. However, whether theses suspected factors have played a role in childhood cancer still needs to be further confirmed.

Air pollution has been a big problem in China over recent years [23], China is one of the countries with highest PM_2.5_ concentration in the world, and annual average PM_2.5_ concentration in Central-Eastern China has been over 100mug/m^3^ in the past two decades [24], especially eastern provinces with higher GDP and population density where the most amount of PM2.5 was emitted in China from 2005 to 2014 [25], similar in other Asian countries such as India [26] and Thailand [27]. Exposure to particular matters (e.g., PM_2.5_) in the smog/haze is considered to be related with the incidence of childhood cancer [28]. However, the number of childhood cancer cases was too small to detect any meaningful trends, so the suspected link between air pollution and childhood cancer was not supported by our findings. To establish the relationship between air pollution and childhood cancer, advanced study designs (e.g., prospective cohort study and spatiotemporal modelling) are required. In consideration of age group, the diagnosis of childhood cancer under the age of 5 was mainly due to heredity whereas the diagnosis of childhood cancer at the ages of 5 or over was regarded to be associated with environmental exposure [29].

Moreover, gender and living regions were not significantly associated with survival rate of childhood cancer. The 5-year survival rate for all childhood cancers was 66.7%, higher than 55.7% reported by Zheng et al. in Shanghai during 2002–2005 [30, 31] and 47.2% in Thailand during 2001–2011 [32], but lower than 71.9% from 198 registries in 53 countries [33].

This study has a number of strengths. We focused on Pudong because it has been a rapidly developing region and socioeconomic development has been unprecedentedly fast over the past decades. In this study, we provide novel insight into childhood cancer incidence rates, patient survival rates, and overall trend of childhood cancer incidence in Pudong. Additionally, a sophisticated statistical approach (i.e., multivariate Cox regression model) was applied to analyze survival risk factors. On the other hand, this study also has several limitations. Only permanent residents in Pudong were included in the cancer registry. In fact, there is a large floating population in Pudong. Taking the population under 15 in 2015 as an example, the number of permanent residents was 157,797 whereas the number of floating population who had residence permit was 320,692. Focusing solely on permanent residents may introduce bias as some childhood cancer patients may not registered in Pudong due to frequent and unstable population exchange. Moreover, due to limited number of cases, the trend analysis was not very useful for several cancer type, and the subtypes of leukemia and CNS tumors were not analyzed.

In summary, this study described the overall cancer incidence in children and the characteristics of childhood cancer in Pudong between 2002 and 2015. We also examined the trends of childhood cancer and explored any changes in survival rates for children with cancer. We found that the most common cancers that occurred in children were leukemia and CNS tumors, the incidence and survival rate for childhood cancer patients varied by age and cancer type, and the overall trends of childhood cancer incidence remained relatively stable from 2002 to 2015. During the recent decades, many advances have been achieved in the treatment of childhood cancer whereas the research in causes and prevention of childhood cancer has lagged behind. Concerted efforts are required for establishing a more-sophisticated cancer registry and identifying causal/risk factors of childhood cancer. Appropriate collaboration is needed to develop effective childhood cancer prevention programs at regional, national and international levels.

## Conclusions

The overall trends of childhood cancer incidence remained relatively stable in Pudong from 2002 to 2015 even though socioeconomic development has been unprecedentedly fast in this region.

## Data Availability

All relevant data is within the paper. The data of children cancer was from Pudong Cancer Registry and Follow-up system, Statistics Bureau and the Public Security Bureau of Pudong. The datasets used in the current study are available from the corresponding author on reasonable request.

## Abbreviations

CNS: central nervous system
SEER: Surveillance, Epidemiology, and End Results
GDP: gross domestic product
RMB: Ren Min Bi (Chinese currency unit)
ICCC-3: International Classification of Childhood Cancer
IARC/IACR: International Agency for Research on Cancer/International Association of Cancer Registries
IICC: International Incidence of Childhood Cancer
ASR: age-standardized rate
TX: Texas
MD: Maryland
